# Right Heart Structural Changes Are Independently Associated with Exercise Capacity in Non-Severe COPD

**DOI:** 10.1371/journal.pone.0029069

**Published:** 2011-12-29

**Authors:** Michael J. Cuttica, Sanjiv J. Shah, Sharon R. Rosenberg, Randy Orr, Lauren Beussink, Jane E. Dematte, Lewis J. Smith, Ravi Kalhan

**Affiliations:** 1 Pulmonary Hypertension Program, Division of Pulmonary and Critical Care Medicine, Northwestern University, Chicago, Illinois, United States of America; 2 Asthma and COPD Program, Division of Pulmonary and Critical Care Medicine, Northwestern University, Chicago, Illinois, United States of America; 3 Division of Cardiology, Department of Medicine, Northwestern University, Chicago, Illinois, United States of America; University of Giessen Lung Center, Germany

## Abstract

**Background:**

Pulmonary hypertension (PH) occurs frequently and results in functional limitation in advanced COPD. Data regarding the functional consequence of PH in less severe COPD are limited. Whether echocardiographic evidence of right sided heart pathology is associated with functional outcomes in patients with non-severe COPD is unknown.

**Methods:**

We evaluated pulmonary function, six minute walk distance, and echocardiography in 74 consecutive patients with non-severe COPD. We performed multivariable linear regression to evaluate the association between right heart echocardiographic parameters and six minute walk distance adjusting for lung function, age, sex, race, and BMI.

**Main Results:**

The mean six minute walk distance was 324±106 meters. All subjects had preserved left ventricular (LV) systolic function (LV ejection fraction 62.3%±6.1%). 54.1% had evidence of some degree of diastolic dysfunction. 17.6% of subjects had evidence of right ventricular enlargement and 36.5% had right atrial enlargement. In univariate analysis RV wall thickness (β = −68.6; p = 0.002), log right atrial area (β = −297.9; p = 0.004), LV mass index (β = −1.3; p = 0.03), E/E' ratio (β = −5.5; p = 0.02), and degree of diastolic dysfunction (β = −42.8; p = 0.006) were associated with six minute walk distance. After adjustment for co-variables, the associations between right atrial area (log right atrial area β = −349.8; p = 0.003) and right ventricular wall thickness (β = −43.8; p = 0.04) with lower six minute walk distance remained significant independent of forced expiratory volume in one second (FEV1). LV mass index, E/E' ratio, and degree of diastolic dysfunction were not independent predictors of six minute walk distance.

**Conclusion:**

In patients with non-severe COPD right sided cardiac structural changes are associated with lower six minute walk distance independent of lung function. These findings may indicate that echocardiographic evidence of pulmonary hypertension is present in patients with non-severe COPD and has important functional consequences.

## Introduction

Chronic obstructive pulmonary disease (COPD) recently surpassed cerebrovascular disease to become the third leading cause of death in the United States, and is the only leading cause of death that is increasing in prevalence [1]. COPD is a complex disease that has effects not only on the lungs but multiple organ systems throughout the body [2], [3], [4]. Current therapy for patients with COPD, including bronchodilators and inhaled corticosteroids, do not modify disease progression [5]. The next innovation in the management of patients with COPD will be identifying and grouping key elements of the clinical presentation into distinct phenotypes to add useful prognostic information and guide more targeted therapy. Identifying these phenotypes early in the course of the disease may allow for effective intervention that will alter the natural history of the disease [6], [7].

Pulmonary hypertension (PH) occurs frequently in patients with advanced COPD and has been associated with decreased exercise capacity and increased mortality independent of degree of impairment in lung function [8], [9]. Given the unique contribution to outcome, PH in COPD appears to represent a distinct phenotype. PH is defined hemodynamically by right heart catheterization [10]. Due to the invasive nature of cardiac catheterization, most prior descriptions of PH in COPD have studied patients with advanced COPD awaiting lung transplantation, in whom cardiac catheterization was clinically indicated [11], [12]. Patients with mild to moderate lung disease do not typically undergo right heart catheterization and as a result PH and its potential functional impact are poorly described in this population. There is, however, evidence that pathologic changes to the pulmonary vasculature consistent with pulmonary hypertension occur at all stages of COPD severity [13].

Echocardiography plays an important role in screening for and following the progression of patients with PH [14], [15]. Although limitations exist in using echocardiography to definitively diagnose PH, it is an indispensible tool in evaluating and following the structural and functional changes of the right heart related to pulmonary hypertension. We investigated if structural changes of the right heart described in patients with pulmonary hypertension are associated with worse exercise capacity in patients with mild, moderate, and moderately severe COPD.

## Methods

We reviewed 74 consecutive patients with clinically indicated echocardiograms and pulmonary function data seen in the Northwestern Asthma and COPD program between July 1, 2006 and April 1, 2010. The Northwestern University Institutional Review Board approved the study and granted a waiver of informed consent. Data collected included general demographic information, six minute walk distance, Modified Medical Research Council (MMRC) dyspnea scores, BODE (Body mass index, degree of Obstruction (FEV_1_), Dyspnea score (MMRC scale), Exercise capacity (six minute walk distance)) index, echocardiographic parameters, and full pulmonary function testing.

Pulmonary function testing including spirometry, lung volumes, and diffusion capacity for carbon monoxide (DLCO) was performed in the hospital pulmonary function laboratory by trained technicians according to ATS-ERS standards and guidelines [16], [17], [18]. We selected patients with mild, moderate, and moderately severe COPD based on guidelines of the American Thoracic Society: mild was defined as FEV1/FVC ratio <0.7 and FEV1 percent predicted >70%, moderate was defined as FEV1/FVC ratio <0.7 and FEV1 percent predicted 60–69%, and moderately severe was defined as FEV1/FVC ratio <0.7 and FEV1 percent predicted 50–59% [19]. Lung volumes were measured using body plethysmography in 95% (70/74) of patients, the remaining 5% were measured by nitrogen washout.

Six minute walk testing was performed in the Northwestern Asthma and COPD clinic by trained technicians according to ATS guidelines [20].

Echocardiograms were performed in the clinical echocardiography laboratory using either Philips (ie33) or GE (Vivid7) echocardiography machines. All studies were analyzed and measured using a systematic protocol by a single cardiologist blinded to all other data. Echocardiographic data collected for analysis included: left ventricular (LV) end diastolic dimension, biplane LV end-diastolic volume, biplane LV end-systolic volume, LV mass, LV ejection fraction, cardiac output, left atrial volume, right ventricular (RV) basilar diameter, RV longitudinal dimension, RV end diastolic area, RV wall thickness, RV outflow track diameter, right atrial area, early mitral inflow velocity (E), late mitral inflow velocity (A), early septal diastolic longitudinal velocity (E'), diastolic function grade, peak tricuspid regurgitation (TR) velocity, pulmonary artery systolic pressure, tricuspid annular plane systolic excursion (TAPSE), and RV fractional area change [21], [22].

All echocardiographic parameters were measured and calculated according to previously published guidelines [21]. Diastolic function was graded by analyzing mitral inflow patterns, tissue Doppler E' velocity, and echocardiographic estimation of LV filling pressure (E/E' ratio) using a previously published method [23]. Patients were categorized as indeterminate diastolic function if they could not be classified based on the aforementioned diastolic function parameters.

Data are presented as mean ± standard deviation or frequency and percent. Univariable and multivariable analyses were performed to evaluate whether echocardiogram parameters or lung function variables predicted six minute walk distance. Comparison of means was done via Student's t tests. Multiple means were compared using analysis of variance (ANOVA) with Bonferroni adjustment for multiple comparisons. The relationships between given parameters and the 6MWD were assessed by univariable and multivariable linear regression. Co-variables included in statistical models were pre-specified based on a known or hypothesized association with exercise tolerance in COPD. Age, sex, height and weight were specifically included as co-variables given their direct impact on six minute walk distance [24]. Statistical analysis was completed using Graphpad software (Prism 4, San Diego, California) and STATA software (STATA 10, College Station, Texas).

## Results

Subjects were predominantly Caucasian (72.2%) and had a mean age of 72±11 years and a body mass index of 27.8±6.0 kg/m^2^. There was no difference in sex, age, or race across levels of COPD severity. The total lung capacity was within normal limits (TLC% predicted; 100±14.7%) but the residual volume was elevated (RV %predicted; 125.9±32.7%). The diffusion capacity for carbon monoxide (DLCO % predicted) was moderately reduced (55.8±18.2%), however oxygen saturation at rest was normal (96.0±2.7%). The mean six minute walk distance was 324±106 meters. The mean six minute walk distance decreased with worsening lung function (mild; 360±109 m, moderate; 333±95 m, moderately severe 288±108 m, p = 0.06) but did not meet statistical significance. The mean MMRC dyspnea score was 1.7±1.1, and mean BODE index was 2.4±2.1. Both MMRC (mild; 1.2±1.3, moderate; 1.8±0.8, moderately severe; 2.0±1.2, p = 0.12) and BODE (mild; 1.6±2.3, moderate; 2.2±1.7, moderately severe; 3.3±2.3, p = 0.04) increased with worsening lung function but only BODE was statistically significant. (**Table 1**)

**Table 1:** Patient Characteristics.

Variable	Total	Mild	Moderate	Moderately Severe	p value
N	74	19	30	25	
Gender (male)	54.1%	63.2%	46.7%	56.0%	0.5
Age	72±11	72±11	72±12	73±10	0.9
Body Mass Index (kg/m^2^)	27.8±6.0	27.8±4.6	28.5±7.2	27.0±5.6	0.7
Race; % (n)					0.4
White	72.2 (52)	79.0 (15)	73.3 (22)	65.2 (15)	
Black	20.8 (15)	15.8 (3)	16.7 (5)	30.4 (7)	
Hispanic	1.4 (1)	5.3 (1)	–	–	
Asian	5.6 (4)	–	10.0 (3)	4.4 (1)	
Lung Function:					
FVC (%)	76.6±13.1	91.1±13.7	75.6±7.7	66.8±6.7	<0.0001
FEV_1_ (%)	64.8±11.7	80.6±10.4	63.7±2.6	54.1±2.8	<0.0001
FEV1/FVC ratio	0.62±0.05	0.64±0.04	0.63±0.05	0.60±0.05	0.01
TLC (%)	100.3±14.7	106.2±12.3	100.4±16.1	96.1±13.5	0.09
FRC (%)	112.9±25.1	109.4±20.2	116.4±27.4	110.6±25.4	0.6
RV (%)	125.9±32.7	118.2±28.4	129.2±36.6	127.3±30.8	0.5
DLCO (%)	55.8±18.2	63.9±20.6	59.3±17.8	45.6±12.0	0.002
O_2_ Saturation (%)	96.0±2.7	96.5±1.1	96.3±1.9	95.3±4.0	0.3
Functional Test:					
6MWD (m)	324±106	360±109	333±95	288±108	0.06
MMRC score	1.7±1.1	1.2±1.3	1.8±0.8	2.0±1.2	0.12
BODE index	2.4±2.1	1.6±2.3	2.2±1.7	3.3±2.3	0.04

FVC; forced vital capacity, FEV_1_; forced expiratory volume in 1 second, ratio; FEV_1_/FVC ratio, TLC; total lung capacity, FRC; functional residual capacity, RV; residual volume, DLCO; diffusing capacity of carbon monoxide, 6MWD; six minute walk distance, MMRC; modified medical research council, BODE; Body mass index, degree of Obstruction (FEV_1_), Dyspnea score (MMRC scale), Exercise capacity (six minute walk distance).

Full assessment of right heart dimensions was available in all 74 subjects. The mean right ventricular basilar diameter (3.7±0.5 cm), right ventricular longitudinal dimension (7.7±0.9 cm) and right ventricular end diastolic area (23.7±4.5 cm^2^) were within normal limits. However, approximately 17.6% of subjects had evidence of right ventricular enlargement [21]. The mean right atrial area was at the upper limit of normal (17.8±5.1 cm^2^) with 36.5% of subjects demonstrating evidence of right atrial enlargement [22]. Right ventricular wall thickness measurements were available in 61 subjects. The mean wall thickness was 4.8±0.6 mm, with approximately 16.4% of subjects demonstrating evidence of right ventricular hypertrophy. The right ventricular outflow tract was noted to be dilated in both the proximal (3.5±0.4 cm) and distal (3.4±0.4 cm) diameters. The mean TR jet velocity (3.0±0.6 m/s) and the pulmonary artery systolic pressure (44.1±16.1 mmHg) were both elevated but measurements were available in only 46 subjects. Right ventricular function as assessed by both TAPSE (2.1±0.5 cm) and right ventricular fractional area change (47.2%±6.3%) was normal. There were no differences in right heart structure parameters, pressure estimates, or right ventricular function when compared across degrees of COPD severity. (**Table 2**)

**Table 2:** Right Heart Parameters by COPD Severity.

Parameter	Total(n = 74)	Mild(n = 19)	Moderate(n = 30)	Moderately Severe(n = 25)	p value*
RV basilar diameter (cm)	3.7±0.5	3.5±0.4	3.8±0.5	3.8±0.6	0.1
RV longitudinal dimension (cm)	7.7±0.9	7.8±0.9	7.7±1.0	7.6±0.9	0.6
RV end diastolic area (cm^2^)	23.7±4.5	22.4±3.9	23.7±4.2	24.6±5.3	0.3
RV wall thickness (mm)	4.8±0.6	4.6±0.5	4.8±0.5	4.9±0.8	0.3
RV outflow track (distal) (cm)	3.4±0.4	3.4±0.4	3.5±0.4	3.4±0.4	0.6
RV outflow track (proximal)(cm)	3.5±0.4	3.4±0.4	3.5±0.5	3.5±0.4	0.8
Right Atrial Area (cm^2^)	17.8±5.1	16.1±2.7	17.8±5.0	19.1±6.2	0.14
TR peak jet velocity (m/s)	3.0±0.6	3.0±0.6	3.0±0.6	3.1±0.6	0.7
PASP (mmHg)	44.1±16.1	42.2±16.3	42.8±15.1	46.7±17.6	0.7
TAPSE (cm)	2.1±0.5	2.2±0.4	2.1±0.5	2.1±0.5	0.6
RV fractional area change (%)	47.2±6.3	47.3±5.5	47.1±5.4	47.4±7.9	0.9

RV; right ventricular, TR; tricuspid regurgitant jet peak velocity, PASP; pulmonary artery systolic pressure, TAPSE; tricuspid annular plane systolic excursion.

*ANOVA comparing mild, moderate, moderately severe.

To better understand the etiology of right heart changes in this patient population a full assessment of left ventricular systolic and diastolic function was obtained. Overall, patients had preserved LV systolic function (LV ejection fraction 62.3%±6.1%) and preserved cardiac output (as calculated by echocardiogram; 4.7±2.5 L/min). The left ventricular end systolic (13.9±3.8 mL/m^2^) and end diastolic (36.7±7.1 mL/m^2^) volume indices were within normal limits. The left ventricular mass index was normal in both males (74.1±19.9 g/m^2^) and females (68.2±20.7 g/m^2^). The left atrial volume indices were at the upper limits of normal for both males (29.4±13.1 mL/m^2^) and females (27.6±10.9 mL/m^2^). There was no clinically significant difference in either LV structural parameters or LV function when compared across degrees of COPD severity. (**Table 3**) Diastolic dysfunction was present in a large percentage of patients: 15 (20.3%) had normal diastolic function, 40 (54.1%) had either mild (9 (12.2%)), moderate (30 (40.5%)), or severe (1 (1.4%)) diastolic dysfunction. The remaining 19 (25.7%) patients were categorized as indeterminate diastolic function due to missing or conflicting diastolic function data. RV structural changes were noted to be present in the sub-group of patients with normal diastolic function. Approximately 6.7% (1/15) were noted to have RV enlargement with RV end diastolic area of greater than 29 cm^2^ and 33.3% (5/15) had right atrial enlargement with right atrial area greater than 18 cm^2^. The RV wall thickness was within normal limits in all patients with normal diastolic function. The degree of diastolic dysfunction was not associated with extent of COPD severity. (Table 3) There was no difference between patients with normal, abnormal (mild, moderate, and severe), or indeterminate diastolic function based on gender, age, body mass index or race. Likewise, lung function and oxygenation did not differ across the diastolic function groups. (**Table 4**)

**Table 3:** Left Heart Parameters by COPD Severity.

Parameter	Total(n = 74)	Mild(n = 19)	Moderate(n = 30)	Moderately Severe(n = 25)	p value*
LV ejection fraction (%)	62.3±6.1	62.9±6.1	60.0±4.6	64.5±5.9	0.01
Cardiac Output (L/min)	4.7±2.5	4.9±2.1	4.0±2.5	5.6±2.6	0.06
LV end systolic volume index (mL/m^2^)	13.9±3.8	13.7±3.2	15.2±4.2	12.4±3.1	0.01
LV end diastolic volume index (mL/m^2^)	36.7±7.1	36.8±4.6	38.1±9.2	34.8±5.3	0.2
LV mass index (g/m^2^):					
Male	74.1±19.9	72.2±18.7	75.7±17.4	74.0±24.1	0.9
Female	68.2±20.7	71.9±27.5	70.1±21.4	62.9±15.0	0.5
LA volume index (mL/m^2^):					
Male	29.4±13.1	27.2±11.5	31.2±14.0	29.5±14.2	0.7
Female	27.6±10.9	26.3±5.4	31.8±14.0	22.4±4.8	0.08
Diastolic Function, n (%)					0.3
Normal	15 (20.3)	5 (26.3)	8 (26.7)	2 (8.0)	
Mild	9 (12.2)	2 (10.5)	2 (6.7)	5 (20.0)	
Moderate	30 (40.5)	8 (42.1)	9 (30.0)	13 (52.0)	
Severe	1 (1.4)	0 (0)	1 (3.3)	0 (0)	
Indeterminate	19 (25.7)	4 (21.1)	10 (33.3)	5 (20.0)	

LV; left ventricular, LA; left atrial.

*ANOVA comparing mild, moderate, moderately severe.

**Table 4:** Comparison of Diastolic Function.

Parameter	Normal Diastolic Function(n = 14)	Abnormal Diastolic Function(n = 40)	Indeterminate Diastolic Function(n = 19)	p value
Gender (male)	60.0%	50.0%	57.9%	0.7
Age	69±12	72±11	74±11	0.3
BMI	25.7±2.7	27.9±6.1	29.4±7.2	0.2
Race; (%)				0.1
Caucasian	12 (80.0)	24 (61.5)	16 (88.9)	
Black	1 (6.7)	12 (30.8)	2 (11.1)	
Hispanic	0 (0)	1 (2.6)	0 (0)	
Asian	2 (13.3)	2 (5.1)	0 (0)	
Lung Function				
FVC (% predicted)	81.8±12.7	75.2±13.4	75.5±12.7	0.2
FEV1 (% predicted)	66.1±8.9	63.7±12.6	66.1±11.8	0.7
FEV1/FVC ratio	0.60±0.05	0.63±0.05	0.64±0.05	0.2
TLC (% predicted)	102.9±14.3	100.0±15.5	99.2±13.7	0.7
RV (% predicted)	120.6±33.4	129.3±36.9	122.8±22.3	0.6
DLCO (% predicted)	58.8±19.3	53.7±18.3	57.8±17.7	0.6
O2 saturation (%)	96±2	96±3	96±2	0.9

BMI; body mass index, FVC; forced vital capacity, FEV1; forced expiratory volume in one second, Ratio; FEV1/FVC ratio, TLC; total lung capacity, RV; residual volume, DLCO; diffusion capacity of carbon monoxide, O2; oxygen.

**Figure 1:**
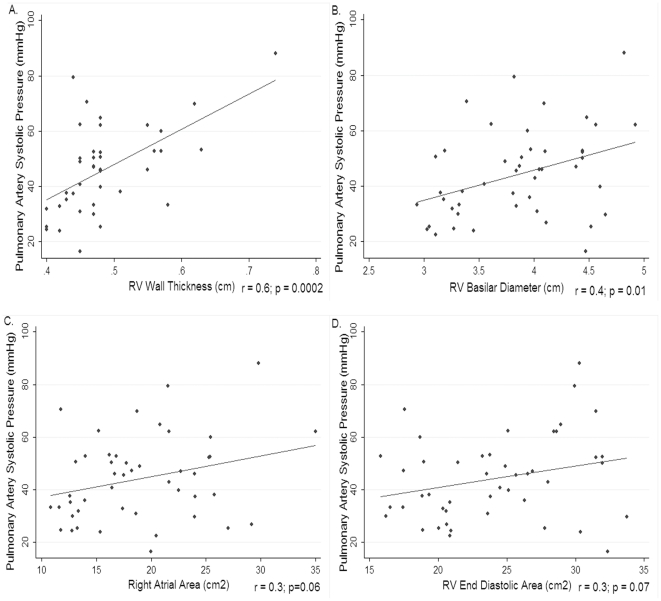
The correlation between right ventricular echocardiographic parameters and pulmonary artery systolic pressure estimates. Panel 1A: The correlation between pulmonary artery systolic pressure (mmHg) and right ventricular wall thickness (cm). Panel 1B: The correlation between pulmonary artery systolic pressure (mmHg) and right ventricular basilar diameter (cm). Panel 1C: The correlation between pulmonary artery systolic pressure (mmHg) and right atrial area (cm^2^). Panel 1D: The correlation between pulmonary artery systolic pressure (mmHg) and right ventricular end diastolic area (cm^2^).

The RV echo parameters correlated with estimated pulmonary artery systolic pressure. RV wall thickness (r = 0.6, p = 0.0002) and RV basilar diameter (r = 0.4, p = 0.01) both correlated with pulmonary artery systolic pressure. The right atrial area (r = 0.3, p = 0.06) and RV end diastolic area (r = 0.3, p = 0.07) both trended toward an association but did not reach statistical significance. Markers of LV diastolic function did not correlate with RV parameters as strongly as PASP: E/A ratio was not correlated with RV end diastolic area (r = 0.003, p = 0.9), right atrial area (r = 0.2, p = 0.1) or RV wall thickness (r = 0.2, p = 0.2). E/E' ratio likewise showed no correlation with RV end diastolic area (r = 0.007, p = 0.9) or right atrial area (r = 0.2, p = 0.4) but did correlate with RV wall thickness (r = 0.3, p = 0.045). (**Figure 1**)

**Figure 2:**
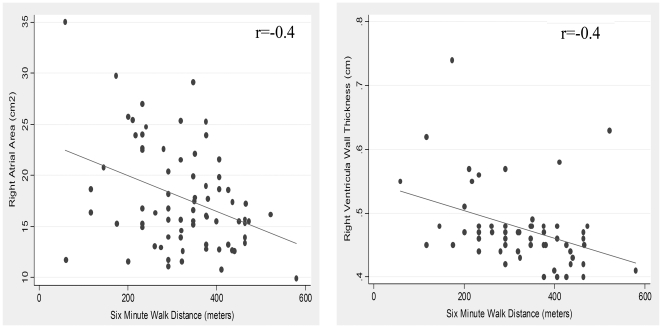
The correlation between right ventricular echocardiographic parameters and six minute walk distance. Panel 2A: The correlation between right atrial area (cm^2^) and six minute walk distance (meters); (r = −0.4, p = 0.002). Panel 2B: The correlation between right ventricular wall thickness (cm) and six minute walk distance (meters); (r = −0.4, p = 0.002).

An evaluation of the association between cardiac function and structural changes on echocardiogram and six minute walk distance independent of lung function impairment was performed using multivariable linear regression. Significant correlations were noted between the six minute walk distance and both right atrial area (r = −0.4, p = 0.002) and right ventricular wall thickness (r = −0.4, p = 0.002). (**Figure 2**) In univariable analysis a 1 mm change in right ventricular wall thickness was associated with a 68.6 meter decline in the six minute walk distance (β = −68.6 m; p<0.002). The log right atrial area (β = −297.9 m; p = 0.004), TR peak jet velocity (β = −0.56 m; p = 0.04), pulmonary artery systolic pressure (β = −2.0 m; p = 0.04), LV mass index (β = −1.3 m; p = 0.03), E/E' ratio (β = −5.5 m; p<0.01) and degree of diastolic dysfunction (β = −42.8 m; p = 0.006) were also inversely associated with six minute walk distance. FEV1 (β = 77.1 m; p<0.001) was associated with six minute walk distance. No association was noted between other right and left heart parameters, and six minute walk distance. (**Table 5**) In multivariable analysis a 1 mm change in right ventricular wall thickness was associated with a 43.8 meter decline in the six minute walk distance (β = −43.8 m; p = 0.04). This association was independent of lung function (FEV1), age, sex, race and BMI. Right atrial area (logRAA; β = −349.8 m; p = 0.003) was also associated with six minute walk distance independent of lung function, age, sex, race and BMI. TR peak jet velocity (β = −0.26 m; p = 0.3), PASP (β = −1.0 m; p = 0.3), LV mass index (β = −0.75 m; p = 0.2), E/E' ratio (β = −2.6 m; p = 0.3) and the degree of diastolic dysfunction (β = −30.1 m; p = 0.06), were not independent predictors of six minute walk distance (**Table 5**).

**Table 5:** Association Between Echocardiogram Parameters and Six Minute Walk Distance.

	Univariate Model		Multivariate Model*	
Echo Parameter	β	p value	β	p value
RV basilar diameter (cm)	−44.2	0.06	–	–
RV longitudinal dimension (cm)	4.8	0.7	–	–
RV end diastolic area (cm^2^)	−1.7	0.5	–	–
RV wall thickness (mm)	−68.6	0.002	−43.8	0.04
RV outflow track (distal) (cm)	−42.2	0.2	–	–
RV outflow track (proximal)(cm)	−35.8	0.2	–	–
log Right Atrial Area (cm^2^)	−297.9	0.004	−349.8	0.003
TR peak jet velocity (m/s)	−0.56	0.04	−0.26	0.3
PASP (mmHg)	−2.0	0.04	−1.0	0.3
TAPSE (cm)	21.5	0.4	–	–
RV fractional area change (%)	308.8	0.12	–	–
LV ejection fraction (%)	35.6	0.9	–	–
Cardiac Output (L/min)	0.001	0.8	–	–
LV end systolic volume index (mL/m^2^)	2.3	0.5	–	–
LV end diastolic volume index (mL/m^2^)	2.2	0.2	–	–
LV mass index (g/m^2^)	−1.3	0.03	−0.75	0.2
LA volume index (mL/m^2^)	−1.9	0.06	–	–
E/A ratio	−6.0	0.8	–	–
E/E' ratio	−5.5	0.02	−2.6	0.3
Diastolic Function	−42.8	0.006	−30.1	0.06

*Covariates: FEV1, Age, Sex, Body Mass Index (BMI), RaceRV; right ventricular, TR; tricuspid regurgitant, PASP; pulmonary artery systolic pressure, TAPSE; tricuspid annular plane systolic excursion, LV; left ventricular.

No association was seen between the echocardiogram parameters and MMRC dyspnea score or the integrative BODE index (data not shown).

## Discussion

We report our experience with cardiac structural changes and their association with patient- centered outcomes in 74 consecutive patients with mild, moderate, or moderately severe COPD. In this cohort of patients without severely impaired lung function we demonstrated that structural changes of the right heart are associated with a decrement in six minute walk distance. This association was independent of the severity of COPD as measured by FEV1 as well as age, sex, race and body mass index. We did not find an association between six minute walk distance and right ventricular function as assessed by TAPSE or left ventricular systolic or diastolic function. This is the first report to our knowledge of right heart structural changes suggestive of pulmonary hypertension in patients with non-severe COPD being linked to a patient-centered functional endpoint. This report is consistent with our findings that pulmonary hypertension of any cause has important functional consequences in patients with advanced COPD, but extends this finding to patients with less severe disease [25].

Pulmonary hypertension is a well described comorbidity in advanced COPD. In a study of close to 5000 patients with advanced COPD awaiting lung transplant, using the standard definition for pulmonary arterial hypertension (WHO Group 1 PAH, [10]) the frequency of PH related to COPD was found to be 30.4% with an additional 17.2% of patients having PH associated with elevated left heart filling pressures or pulmonary venous hypertension. The mean pulmonary artery pressure was found to be an independent predictor of a lower six minute walk distance and was associated with an increased risk of death while awaiting transplant [8]. This highlights the frequency and functional impact of pulmonary vascular abnormalities in patients with advanced lung disease. The prevalence and impact of pulmonary vascular abnormalities in patients with non-severe COPD is unknown.

Pulmonary hypertension is defined hemodynamically by right heart catheterization and therefore in patients with non-severe COPD who are typically not subjected to the risks of catheterization pulmonary hypertension is not well described. Higham et al. evaluated 73 consecutive COPD patients in an outpatient setting with echocardiographic estimates of pulmonary artery pressures and found PH to be present in 25% of patients with mild COPD and 43% of those with moderate COPD [26]. Although limitations exist in using echocardiography to definitively diagnose PH, this tool plays an important role in screening for and following the progression of patients with all forms of PH [14], [15]. The utility of echocardiography has been particularly questioned in patients with COPD as air trapping and lung hyperinflation may make attaining adequate ultrasound windows difficult. In our cohort of patients with less severe lung disease there was no significant hyperinflation and only mild air trapping, and high quality RV structural data were obtained in all 74 patients. Therefore, utilizing non-invasive echocardiogram to explore the importance of the effect of pulmonary vascular changes on the right heart in less severe lung disease is feasible in this population.

We found that right ventricular wall thickness and right atrial area were associated with lower six minute walk distance independent of lung function. Both right ventricular wall thickness and right atrial area play an important prognostic role in various forms of pulmonary hypertension [27], [28], [29]. Enlargement of the right atrium is an independent predictor of adverse outcomes in patients with idiopathic pulmonary arterial hypertension [27]. In a separate study increased RV wall thickness was associated with a decreased risk of death in IPAH patients with a dilated right ventricle. Interestingly, this study also demonstrated that the mean pulmonary artery pressure was the only independent predictor of RV wall thickness [29]. In our cohort we found a correlation between the estimated pulmonary artery systolic pressure on echocardiogram and the right ventricular end diastolic area, right atrial area and right ventricular wall thickness. This finding suggests that an elevation in pulmonary artery pressures may play a role in the structural changes described in the right heart of COPD patients. Furthermore, both enlargement of the right atrium and RV hypertrophy have been postulated to be an early indicator of RV dysfunction related to pulmonary hypertension in an at-risk patient population [28]. Interestingly, we did not see an association between either the right ventricular fractional area change or TAPSE (a marker of RV longitudinal systolic function) and six minute walk distance suggesting that perhaps structural changes of the right heart related to pulmonary hypertension may precede functional changes. Although right atrial enlargement and right ventricular wall hypertrophy have been described in patients with mild and moderate COPD their association with patient-centered functional endpoints have not been explored [30].

The subjects in this study had preserved left ventricular systolic function but, a large percentage (56.3%) exhibited some degree of diastolic dysfunction. Although it is clear that the right heart structural changes seen in this study are independent of LV systolic function, without right heart catheterization it is difficult to differentiate if the changes are related to LV diastolic dysfunction or primary pulmonary vascular disease. It is interesting to note that structural changes of the right heart were present in the subset of patients with both normal left ventricular systolic and diastolic function. Unfortunately, the total number of observations (n = 15) is too small to draw conclusions related to their impact on outcomes in this subset of subjects. In advanced COPD the functional impact of pulmonary hypertension appears to be independent of the distinction between PH related to pulmonary vascular disease and PH related to diastolic heart failure. The mean pulmonary artery pressure has been shown to be associated with a lower six minute walk distance independent of the left heart filling pressures which likely drive pulmonary hypertension related to diastolic heart failure. Furthermore, both primary pulmonary vascular disease and pulmonary venous hypertension related to diastolic heart dysfunction impacted survival of patients with COPD on the lung transplant list compared to those with normal hemodynamics [25]. However, we know that the therapeutic implications of the distinction between pulmonary arterial hypertension (WHO group 1) and pulmonary venous hypertension (WHO group 2) are important. Trials looking at PH specific therapies in COPD have generally been negative perhaps in part related to the heterogeneity of PH related to COPD. Therefore, although the mortality and functional impact of PH in COPD may be independent of the type of PH, the distinction may be very important when considering therapies to address PH in COPD whether they be further trials of pulmonary vasodilators or other novel interventions.

There are a number of limitations to our study. Since echocardiography was used instead of cardiac catheterization it is not possible to clearly distinguish whether these patients had PH related primarily to pulmonary vascular changes or PH related to left heart disease such as left ventricular diastolic dysfunction. However, the association between right heart structure and six minute walk distance remained independent of LV systolic and diastolic dysfunction. These multivariable regression analyses suggest that right heart structural changes, above and beyond that due to left heart disease may be playing a role in limiting exercise capacity in non-severe COPD. Furthermore, previous data have shown that although the distinction between pulmonary arterial and pulmonary venous hypertension is likely important when making therapeutic decisions, the functional implications of PH in COPD may be independent of the underlying cause of PH [8], [31]. Nevertheless, to best characterize these patients a right heart catheterization would be optimal. The reliance on resting echocardiogram to predict exercise tolerance may also limit our findings. Isolated exercise induced PH is common in COPD. One study that prospectively followed 131 subjects with moderate to severe COPD found that 58% (76/131) of subjects had exercise induced PH with normal resting hemodynamics. The presence of isolated exercise induced PH was a predictor of developing resting PH in the 6 year follow up [32]. Therefore, relying only on a resting echocardiogram may underestimate the presence of pulmonary hypertension. We chose to focus on structural changes of the right heart such as RV wall thickness rather that the traditional pressure estimations based on tricuspid regurgitant jet velocity which may help to capture more of the dynamic changes in pulmonary pressures over time. Some assessment of exercise induced cardiac changes, however, would add interesting information to further define PH in non-severe COPD.

In conclusion, we demonstrated that in subjects with non-severe COPD right heart structural changes are associated with reduced six minute walk distance. Early identification of right heart structural changes in COPD may result in identification of a specific COPD phenotype which is associated with reduced exercise capacity. This is consistent with prior reports of PH in advanced COPD having distinct prognostic and functional implications. Identification of cardiac structural changes in patients with COPD that are associated with patient centered outcomes may lead to novel therapeutic targets to impact a specific disease phenotype.
